# HMGA proteins in the nervous system: role in brain tumorigenesis and neurodegeneration

**DOI:** 10.1038/s41420-026-03242-4

**Published:** 2026-07-11

**Authors:** Carolina Canè, Simona Paladino, Andrea Conte, Giovanna Maria Pierantoni

**Affiliations:** 1 Department of Molecular Medicine and Medical Biotechnology, University of Naples Federico II, Naples, Italy; 2 The Azienda Ospedaliera Universitaria AOU, University of Naples Federico II, Naples, Italy

**Keywords:** Neurodegenerative diseases, Cancer

## Abstract

High Mobility Group A (HMGA) proteins function as non-histone chromatin-associated architectural regulators that modulate gene transcription by modifying chromatin structure rather than binding DNA in a sequence-specific manner. By altering chromatin conformation, HMGA proteins coordinate complex transcriptional programs essential in both physiological and pathological conditions. This review highlights their emerging roles in the context of the nervous system, brain tumorigenesis, and neurodegeneration. We discuss how HMGA-mediated chromatin remodeling and transcriptional regulation influence neurodevelopmental processes, regulating neural stem cell proliferation, differentiation, and lineage specification. We also highlight evidence linking HMGA dysregulation to malignant transformation and progression of brain tumors, where these proteins support oncogenic transcriptional networks. Finally, we explore their possible involvement in molecular pathways associated with neurodegenerative disorders. By integrating findings from developmental neurobiology, oncology, and neurodegeneration research, we aim to provide a comprehensive overview of HMGA proteins as pivotal regulators of neural homeostasis and highlights their potential as biomarkers and therapeutic targets in neurological diseases.

## Facts

- HMGA proteins act as non-histone chromatin architectural regulators, modulating gene transcription by altering chromatin structure rather than binding DNA in a sequence-specific manner.
- Dysregulation of HMGA proteins is associated with malignant transformation and progression of brain tumors through support of oncogenic transcriptional networks.
- Emerging evidence links HMGA proteins to molecular pathways involved in neurodegenerative disorders, suggesting a broader role in nervous system pathology.
- HMGA proteins represent potential biomarkers and therapeutic targets in neurological diseases, bridging developmental neurobiology, oncology, and neurodegeneration research.

## Introduction

High Mobility Group A (HMGA) protein family includes two non-histone, chromatin-associated architectural transcriptional factors (TFs), HMGA1 and HMGA2, that play essential roles in the regulation of chromatin dynamics and gene expression [1, 2]. Unlike conventional TFs, HMGA proteins do not modulate transcription per se; instead, they function primarily by altering chromatin architecture to facilitate or inhibit the binding of other transcriptional regulators, and by directly contributing to the recruitment or displacement of TFs and epigenetic modifiers [3]. Their chromatin remodeling ability is a crucial determinant of HMGAs function in physiological and pathological contexts.

## Structure and function of HMGA1 and HMGA2

**Fig. 1:**
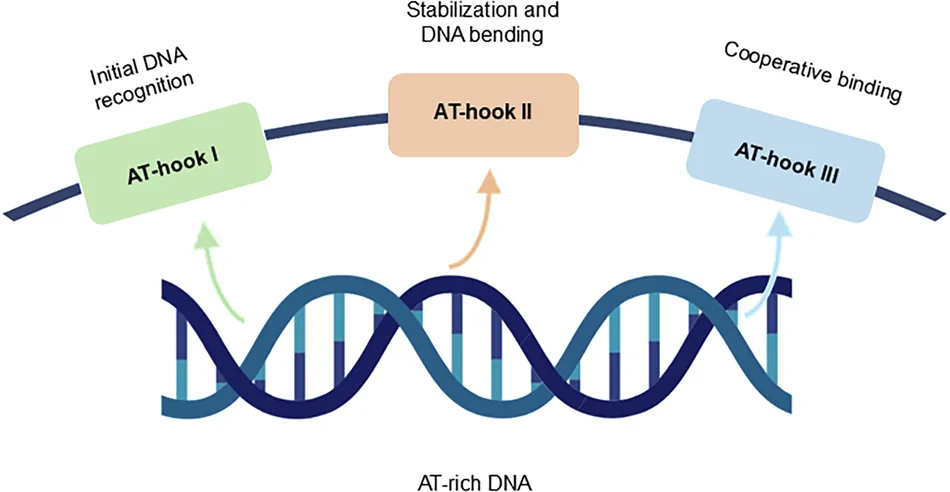
Schematic representation of the three AT-hook motifs in HMGA proteins. Each AT-hook domain contains the conserved RGRP core and binds the minor groove of AT-rich DNA.

Despite being encoded by distinct genes, HMGA1 and HMGA2 share a similar domain architecture, with three conserved N-terminal AT-hooks, which recognize and bind AT-rich regions in the DNA minor groove, and a highly acidic C-terminal tail, involved in the interaction with nuclear proteins, including histones and histone-modifying enzymes [1, 4]. Each AT-hook motif contains an RGRP core motif embedded within a context enriched in positively charged residues that enhance DNA binding and ensure the correct orientation for protein–DNA interactions [5] (Fig. 1). The three motifs are separated by intrinsically disordered linkers, which confer the necessary conformational flexibility for simultaneous or sequential interactions [6, 7].

Collectively, their structure enables HMGA proteins to bend DNA, modulate higher-order chromatin structure, and promote enhanceosome assembly, processes that are particularly relevant in controlling cell fate decisions. Enhanceosomes are multiprotein complexes that include TFs, coactivators, and chromatin remodelers to coordinate gene activation, acting as transcriptional hubs integrating multiple regulatory inputs [8].

Alternative splicing further diversifies HMGA function. In particular, *HMGA1* gene is transcribed into three major splice variants, HMGA1a, HMGA1b, and HMGA1c [1]. HMGA1a and HMGA1b isoforms differ by the inclusion of 11 amino acids present in HMGA1a but absent in HMGA1b, resulting from alternative splicing of a short exon within the C-terminal tail [1, 9, 10]. These two isoforms are the quantitatively dominant HMGA1 species in most adult tissues and tumor samples, and they share the same AT-hook motifs, with differences located exclusively in the intrinsically disordered acidic tail. Importantly, although historically considered functionally redundant, comparative phospho-/acetyl-/methyl-proteomics studies revealed that HMGA1a and HMGA1b accumulate distinct post-translational modifications (PTMs) in vivo, and that these differences correlate with divergent transcriptional outputs and metastatic potential in MCF-7 models [11]. A third splice variant, HMGA1c is currently far less characterized, and its function remains limited and cannot yet be inferred by analogy to HMGA1a/b [12]. The canonical HMGA2 transcript (HMGA2a) consists of exons 1–5 and encodes three AT-hooks followed by a spacer and an acidic C-terminal tail [13]. However, HMGA2 pre-mRNA undergoes alternative splicing, whereby intron-3-derived sequences can function as alternative terminal exons. This process generates multiple endogenous HMGA2 splice variants (HMGA2b–f) that differ in their C-terminal region [13]. Growing evidence from HMGA2 research suggests that loss or alteration of the acidic tail changes the regulatory potential of HMGA2: truncated HMGA2 molecules preserve DNA-binding but have altered interactions with protein partners and yield enhanced transforming outputs in cellular and in vivo systems.

Both *HMGA1* and *HMGA2* genes display tightly regulated expression during embryogenesis, with high levels in proliferating, undifferentiated cells, in which they preserve epigenetic plasticity and orchestrate lineage commitment [14, 15]. By contrast, *HMGA* gene expression is repressed in most adult tissues through transcriptional and post-transcriptional mechanisms, with aberrant reactivation frequently linked to tumorigenesis [16]. Mechanistically, HMGA proteins modulate chromatin accessibility by facilitating enhanceosome assembly and by antagonizing the chromatin-condensing activity of histone H1. This functional interplay links HMGA architectural activity to the temporal control of cell fate decisions. Engagement by HMGA1 and HMGA2 of ATP-dependent remodelers like SWI/SNF complexes further facilitates nucleosome repositioning and enhancer accessibility [17], while cooperation with Polycomb proteins such as EZH2 contributes to the establishment of repressive chromatin states [18].

Crucially, HMGA proteins are central to epigenetic reprogramming. In fact, they modulate histone acetylation, deacetylation, DNA methylation, and nucleosome positioning through interactions with histone acetyltransferases (HATs), histone deacetylase complexes (HDACs), and DNA methyltransferases (DNMTs), as demonstrated in stem cells and tumor models [15]. This property enables stem and progenitor cells, including neural stem/progenitor cells in brain development, to balance self-renewal and lineage commitment [19, 20]. Overall, HMGA proteins represent central hubs of nuclear architecture and transcriptional control that regulate gene expression, cell fate decisions, and oncogenic transformation by influencing TF recruitment, chromatin remodeling, and epigenetic modification.

### HMGA proteins in nervous system development

HMGA proteins play a pivotal role in central and peripheral nervous system (CNS and PNS) development, where their expression is tightly regulated in a temporal and spatial manner [21]. During embryogenesis, HMGA1 and HMGA2 are abundantly expressed in proliferating and undifferentiated cells, including neural crest derivatives and mesenchymal progenitors, thereby sustaining epigenetic plasticity and responsiveness to differentiation cues [22]. In neural stem cells (NSCs), HMGA2 is particularly critical for the maintenance of self-renewal and proliferative ability by repressing cell cycle inhibitors such as p16^Ink4a^ and p19^Arf^. Moreover, HMGA2 orchestrates the temporal dynamics of corticogenesis, influencing the balance between progenitor expansion and neuronal maturation. Consistently, deregulated HMGA2 expression can alter the temporal dynamics of neural progenitor differentiation, leading to abnormal cortical layering [23].

During CNS development, HMGA1 and HMGA2 act as chromatin-architectural proteins that fine-tune lineage specification by modulating TF binding to DNA. In neural stem and precursor cells (NSCs/NPCs), HMGA proteins bind AT-rich regions through their AT-hook domains and compete with linker histone H1, thereby reducing nucleosomal compaction and maintaining a globally open and highly dynamic chromatin configuration. This mechanism, extensively characterized in early cortical NPCs, promotes broad chromatin accessibility to developmental regulators and sustains the neurogenic transcriptional program. Indeed, loss-of-function of HMGA1/2 leads to premature chromatin condensation, repression of neurogenic gene networks, decreased H1 mobility, and an accelerated transition toward glial differentiation; conversely, sustained HMGA expression restores or prolongs the neurogenic potential even in late-stage NPCs [24, 25].

In pluripotent and progenitor contexts, HMGA2 cooperates with several TFs, such as OTX2, during the exit from the naïve pluripotent state: HMGA2 is required for OTX2 recruitment to newly engaged enhancers, enabling the activation of early epiblast- and neuroectoderm- associated genes [26]. More broadly, HMGA proteins function as essential enhanceosome components, stabilizing multi-protein transcriptional complexes and promoting occupancy of TFs throughout CNS development and regulation. These findings highlight the ability of HMGA proteins to cooperate with different transcriptional programs, such as those involving OCT4 [15, 22], NFIA/NFIB [25], NF-κB [22], and STAT-family factors [25]. These TFs regulate interconnected developmental and pathological processes ranging from pluripotency maintenance (OCT4) to gliogenic differentiation (NFIA/NFIB and STAT signaling) and inflammatory responses (NF-κB).

As chromatin transitions from a neurogenic to gliogenic state, HMGA-driven architectural remodeling ensures that NPCs remain competent to respond to stage-specific developmental signals [24].

Through global regulation of chromatin structure and local assembly of transcriptional hubs, HMGA proteins preserve the transcriptional plasticity of progenitor populations and ensure their competence to integrate stage-specific developmental cues. HMGA2 additionally exerts transcriptional control through selective regulation of growth- and fate- associated genes. In early cortical NSCs, HMGA2 directly promotes the expression of Igf2bp2 and Plag1, two regulators of stemness, self-renewal, and metabolic fitness, thereby sustaining proliferative capacity and neurogenic output [25]. These interactions integrate with broader epigenetic transitions characteristic of developing NSCs, including progressive DNA methylation changes, redistribution of H3K27me3 by Polycomb complexes, and chromatin accessibility shifts that affect the NFIA-bound regulatory landscape [25]. In this context, HMGA proteins act as molecular modulators that stabilize the epigenetic configuration of early-stage NSCs while delaying the establishment of the gliogenic chromatin signature.

In the PNS, HMGA proteins similarly influence lineage progression and chromatin structure in Schwann cell precursors, but their function is likely further shaped in the adult nerve by injury-induced growth factor (GF) signaling. Following peripheral nerve injury, Schwann cells undergo extensive transcriptional reprogramming driven by increased levels of NGF, BDNF, GDNF, CNTF, FGF2, and other GFs, which activate PI3K–AKT, MAPK/ERK, JAK/STAT, and cAMP/CREB pathways [27]. These signaling cascades converge on injury-responsive transcriptional regulators, including c-Jun, promoting repression of the pro-myelinating program (e.g., EGR2) while activating a repair-associated gene network. This transition results in widespread chromatin remodeling, downregulation of myelin genes, and induction of genes linked to demyelination and regeneration support, ultimately driving the shift from a myelinating phenotype to a repair Schwann cell state [27]. Given their established role as architectural chromatin regulators, HMGA proteins are well positioned to modulate this large-scale epigenetic reorganization; however, their direct mechanistic contribution to the injury-induced repair program in Schwann cells remains to be fully defined.

Collectively, HMGA proteins emerge as central regulators of neural stem cell biology, integrating cell cycle control, lineage specification, chromatin remodeling, and transcriptional regulation. Their activity ensures the balance between proliferation and differentiation during nervous system development, preserves chromatin plasticity required for neural maturation, and contributes to both physiological homeostasis and disease-associated dysregulation.

### Regulatory mechanisms

The expression and activity of HMGA proteins are regulated by a multilayered network encompassing transcriptional, post-transcriptional, and post-translational mechanisms, ensuring that HMGA levels are precisely modulated during embryogenesis, differentiation, and tissue homeostasis.

At transcriptional level, HMGA gene expression is tightly regulated by a complex interplay of developmental cues, growth factors, and oncogenic signaling pathways, including Wnt/β-catenin [28], TGF-β [29], and PI3K/AKT [30]. These pathways converge on *HMGA1* and *HMGA2* promoters, modulating the recruitment and activity of a range of TFs. The *HMGA* promoters contain multiple regulatory elements that allow integration of diverse signals and are characterized by shared binding domains for specific transcription factors. For example, E-box motifs are recognized by basic helix-loop-helix (bHLH) TFs, and the overexpression of the bHLH factor Hes5 is responsible for an abnormal expression of both *Hmga1* and *Hmga2* in mouse neural cells [31]. Recent evidence has also highlighted the contribution of Sp1 and E2F TFs to the regulation of *HMGA1* expression. In particular, the relative abundance of activating versus repressive E2F family members determines *HMGA1* expression levels, thereby linking *HMGA1* transcriptional control to the balance between quiescent and proliferative states [32]. In addition, Wnt/β-catenin signaling promotes *HMGA2* expression by stabilizing β-catenin, which forms a transcriptional complex with TCF/LEF factors on the *HMGA2* promoter, driving its expression in progenitor and stem cells [33]. Similarly, TGF-β signaling can induce *HMGA2* through SMAD-mediated activation of promoter elements, linking HMGA expression to processes such as tissue remodeling [34].

Several microRNAs (miRNAs) have been identified as critical post-transcriptional regulators of HMGA proteins. For instance, HMGA1 is predicted to be targeted by miRNAs such as miR-296 that specifically represses *HMGA1* expression by promoting the degradation and inhibiting the translation of its mRNA in prostate cancer [35]. Within the high homology regions of the gene, perfectly conserved binding sites were also found for miR-103, miR-142-3p, miR-370, and miR-432, suggesting a potential role for these miRNAs in modulating HMGA1 expression [36].

Additionally, some miRNAs have been experimentally demonstrated to target HMGA1, including miR-15 and miR-16 [37], miR-26a [38], miR-214 [39], miR-548c-3p [40], and miR-761 [36]. These miRNAs primarily act by binding to complementary sequences in HMGA1 mRNA, affecting its stability or translation, and thus represent key components of the post-transcriptional networks that regulate this protein, which is known for its role in chromatin structure and gene transcription regulation. Among these miRNAs, some have also been implicated in neuropathological conditions. For instance, miR-142-3p-whose deregulation has been reported in disorders such as Multiple Sclerosis and Parkinson’s disease has been associated with neuroinflammatory responses and neuronal apoptosis [41], while other HMGA1-regulating miRNAs, including miR-15/16 and miR-26a, as well as miR-103, have been reported to show altered expression in neurodegenerative contexts such as Alzheimer’s disease [42–44], suggesting a potential contribution of HMGA1-related miRNA networks to mechanisms of neurodegeneration.

In parallel, HMGA2 is predominantly regulated by miRNAs of the let-7 family, which target multiple conserved sites within the HMGA2 3′-untranslated region (3′-UTR), leading to mRNA degradation and translational repression [22,45]. Several other miRNAs, including miR-26a, miR-33b, miR-98, and miR-195, have been implicated in controlling HMGA1 and HMGA2 levels in glioblastoma, breast, lung, and hepatocellular carcinoma, underscoring the complexity of post-transcriptional regulation [46]. Notably, isoforms such as let-7b and let-7e have also been implicated in neurodegenerative disorders like Alzheimer’s disease [47], while other HMGA-regulating miRNAs, including miR-98 and miR-195, have been reported to have altered expression in neurodegenerative contexts, further supporting a potential involvement of HMGA-associated miRNA networks in brain pathology [42, 48].

HMGA proteins are extensively regulated at the post-translational level through phosphorylation and acetylation, which modulate their DNA-binding affinity, protein stability, and transcriptional activity [49]. Phosphorylation by kinases such as Casein Kinase 2 (CK2) and Protein Kinase C (PKC) primarily targets the acidic C-terminal tail of HMGA1 and HMGA2, reducing their affinity for AT-rich DNA sequences and altering chromatin association [50]. In particular, CK2-mediated phosphorylation of HMGA1 decreases its DNA-binding capacity and modulates its architectural role within enhanceosomes [51]. Similarly, PKC-dependent phosphorylation negatively affects HMGA-DNA interactions and influences cell cycle progression [50]. In addition to these kinases, the nuclear serine/threonine kinase HIPK2 has been shown to phosphorylate HMGA1 at distinct sites, functionally linking HMGA regulation to stress-response pathways [52]. Unlike CK2 and PKC, whose phosphorylation events mainly modulate DNA-binding affinity, HIPK2-mediated phosphorylation impacts HMGA1 protein stability and transcriptional activity, particularly under genotoxic stress conditions. This modification promotes proteasome-dependent degradation of HMGA1 and functionally connects HMGA1 to p53-dependent apoptotic signaling [53]. Thus, HIPK2 acts as a regulatory node integrating chromatin architecture with DNA damage response pathways. Furthermore, it has also been demonstrated that HMGA1 acetylation by CREB-binding protein (CBP) and p300/CBP-associated factor (PCAF) can affect the stability of enhanceosomes, in which HMGA proteins act as crucial assembly factors [50].

Conversely, methylation of arginine residues by protein arginine methyltransferases (PRMTs) is less understood, and the biological function is still unclear, it seems to be relevant for chromatin remodeling under conditions of cellular stress or apoptosis. The HMGA1b isoform has been reported to bear multiple methyl groups, with four specifically identified through peptide mapping [54]. In addition, recent evidence demonstrated for the first time that HMGA1a is subject to mono-methylation, particularly during apoptosis in leukemic cells. Importantly, this modification occurs independently of phosphorylation status and is consistently detected both in drug-induced and virus-induced apoptotic models, suggesting that HMGA methylation represents a stress-related post-translational event with potential implications for chromatin remodeling and cell fate determination [55, 56].

Furthermore, SUMOylation has emerged as central regulatory mechanism for HMGA2, which has been identified as a bona fide SUMOylation target, despite lacking a canonical SUMO consensus motif. Lysine residues 66 and 67 were shown to be essential for this modification, and SUMOylation-defective mutants fail to undergo conjugation. SUMOylation of HMGA2 promotes the selective downregulation of the promyelocytic leukemia (PML) protein by facilitating its degradation. Mechanistically, SUMOylated HMGA2 recruits ubiquitin-proteasome machinery, leading to PML ubiquitination and subsequent proteasomal degradation. This dual regulation underscores a functional interplay in which SUMOylation acts as a prerequisite for HMGA2-driven ubiquitin-dependent degradation of PML. Importantly, pharmacological agents such as arsenic trioxide enhance HMGA2 SUMOylation and its colocalization with PML nuclear bodies, accelerating PML clearance [57].

In summary, HMGA proteins are regulated by a complex, multilayered network that keeps their expression and activity tightly controlled under normal physiological conditions. Strategies that restore miRNA-mediated repression, block oncogenic upstream signals, or selectively modify HMGA post-translational modifications provide possible ways to correct their dysregulation, with implications for both cancer therapy and regenerative medicine [58, 59].

**Fig. 2:**
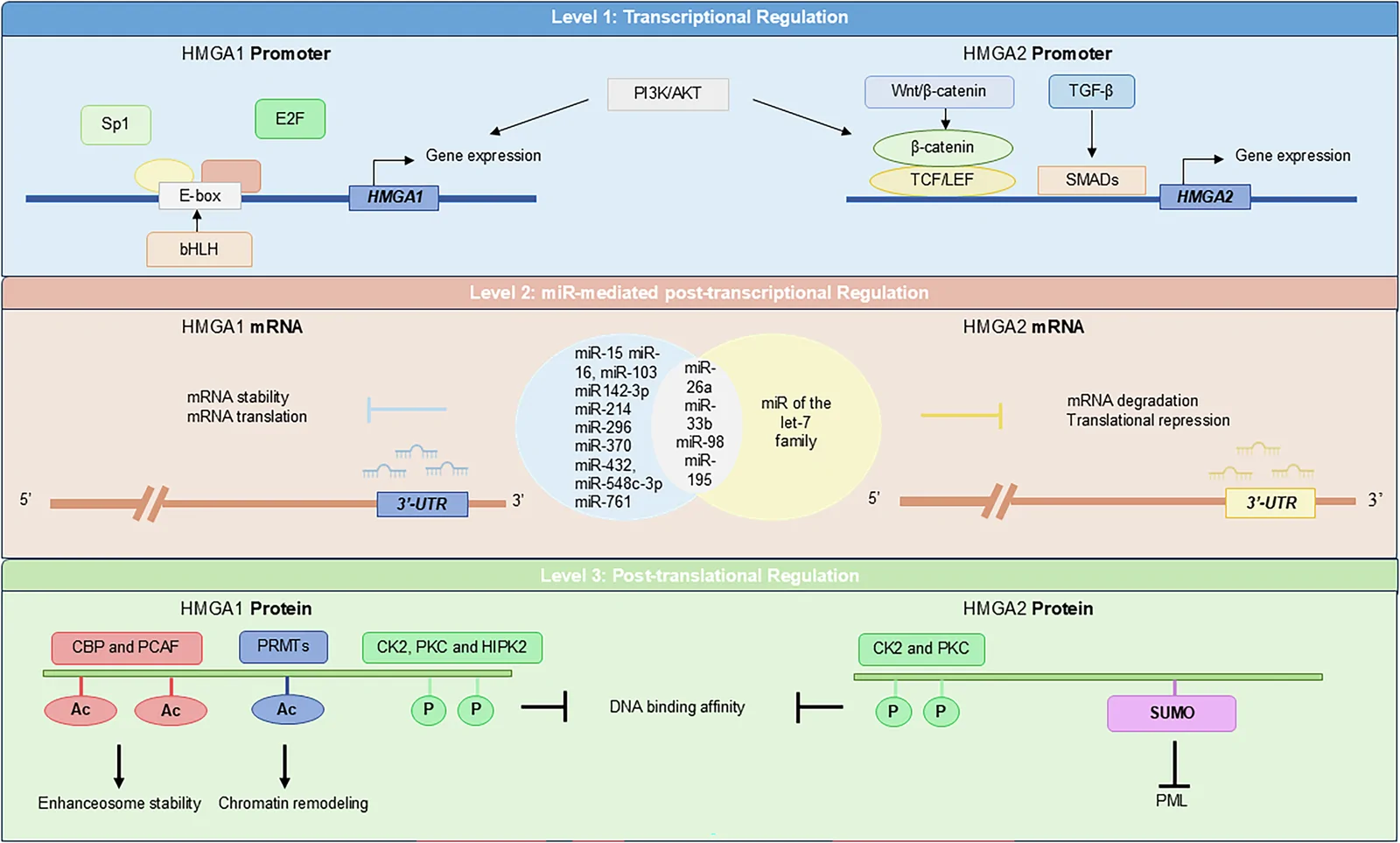
Multilayered control encompassing transcriptional, post-transcriptional and post-translational mechanisms modulating HMGA1 and HMGA2.

The described mechanisms controlling HMGA1 and HMGA2, spanning transcriptional, post-transcriptional, and post-translational levels, are summarized in Fig. 2.

## Hmga expression in brain tumors

The physiological roles of HMGA proteins in stem cell maintenance, lineage specification, and adult neurogenesis suggest their potential relevance in pathogenesis of oncological disorders. In fact, when aberrantly expressed or dysregulated, HMGAs inappropriately reactivate developmental transcriptional programs, driving tumorigenesis and therapy resistance. Consistently, HMGA proteins have been increasingly implicated in the pathogenesis and progression of many cancer types, in which their aberrant expression is a hallmark of aggressive phenotypes and poor clinical outcomes [58, 59]. Here, we will focus on brain tumors, including gliomas, medulloblastomas (MB), and meningiomas.

### Role of HMGA1 and HMGA2 in gliomas

Gliomas represent the most common group of primary brain tumors in adults and encompass a wide range of histological and molecular subtypes, from low-grade astrocytomas to glioblastoma multiforme (GBM), the most aggressive and lethal form. A shared hallmark across the glioma spectrum is the marked overexpression of HMGA1 and HMGA2 compared to normal brain tissue [60, 61]. This dysregulation correlates with increasing tumor grade, enhanced proliferative and invasive potential, and resistance to standard therapies such as radiotherapy and temozolomide [62]. Beyond these tumor cell-intrinsic roles, HMGA1 is increasingly recognized as a critical determinant of tumor–microenvironment (TME) organization. In fact, multi-omics analyses suggest that HMGA1 overexpression is associated with a transcriptional program consistent with an immune-excluded and immunosuppressive TME. In particular, the ESTIMATE (Estimation of STromal and Immune cells in MAlignant Tumor tissues using Expression data) algorithm revealed dysfunctional immune infiltration [63]. In gliomas and other cancers, this phenotype appears to involve enrichment of regulatory T-cell-associated programs, increased expression of immune checkpoint-related signatures, and diminished effector immune activity, collectively contributing to tumor immune escape and disease aggressiveness [63]. These findings suggest that HMGA1 may extend its oncogenic functions beyond tumor cell autonomy to the modulation of the glioma immune ecosystem and resistance to immune-modulating therapies [63].

Analyses of glioma samples, including both astrocytomas and GBM, as well as experimental models, have shown that elevated HMGA1 expression is a negative prognostic marker, supporting malignant phenotypes such as invasion, angiogenesis, and maintenance of stem-like traits [64, 65]. HMGA1 promotes proliferation and survival by acting as a critical mediator of oncogenic signaling. It antagonizes the activity of tumor suppressors, such as p53, and activates pro-survival pathways including PI3K/AKT [66] and MAPK [65], thereby facilitating adaptation to hypoxia, oxidative stress, and DNA damage. Additionally, HMGA1 forms functional complexes with cytoskeletal components like vimentin, which potentiate migration and invasion and are consistent with its association with poor outcomes in GBM patients [66].

The central role of HMGA1 in glioma biology is due to its regulation of glioma stem-like cells (GSCs), a subpopulation essential for tumor initiation, recurrence, and resistance to therapy. HMGA1 acts as a critical downstream effector of tumor-suppressive miRNAs such as miR-1297 and miR-296-5p, whose epigenetic repression leads to HMGA1 overexpression in GSCs, enhancing self-renewal and Sox2-mediated stemness [67]. HMGA2 is likewise enriched in GSCs, particularly in the CD133+ compartment where it sustains self-renewal, invasion, and in vivo tumorigenicity. In fact, HMGA2 silencing resulted in reduced clonogenicity, increased apoptosis, and a significant extension of survival in orthotopic xenograft mouse models. Importantly, these effects were not linked to changes in proliferation rates, but rather to the preservation of a stem-like and pro-invasive phenotype [68].

In addition, HMGA2 has been linked to the invasive phenotype of gliomas through the activation of matrix metallopeptidase 2 (MMP2). Zhang et al. demonstrated that HMGA2 acts as a novel recognition partner of the histone acetyltransferase General Control Non-derepressible 5 (GCN5), recruiting it to AT-rich regions within the *MMP2* promoter. This interaction promotes local acetylation of H3K9ac and H3K12ac, induces long-range chromatin conformational remodeling, and ultimately drives MMP2 transcription. In patient cohorts, HMGA2 expression correlated with glioma grade and predicted poor outcome, while MMP2 levels showed a positive but less precise prognostic association. These findings underscore HMGA2 as a central epigenetic modulator of invasion in malignant gliomas, acting through a GCN5/MMP2 axis [61].

Besides its role as an oncogenic driver overexpressed in tumor cells, HMGA2 also harbors functional germline variants that influence the risk of developing glioma. In particular, Zhou et al. reported that a single nucleotide polymorphism (SNP) in *HMGA2* gene (rs6581658 A > G) significantly increases glioma susceptibility in Chinese children, with carriers of the GG genotype showing more than a threefold risk increase. Stratified analyses further suggested genotype-specific effects across age, sex, tumor subtype, and clinical grade. Together with data on HMGA2 overexpression and downstream effectors, the association of HMGA2 SNPs with glioma risk highlights the multifaceted clinical relevance of HMGA2 dysregulation in brain tumor biology [69].

**Fig. 3:**
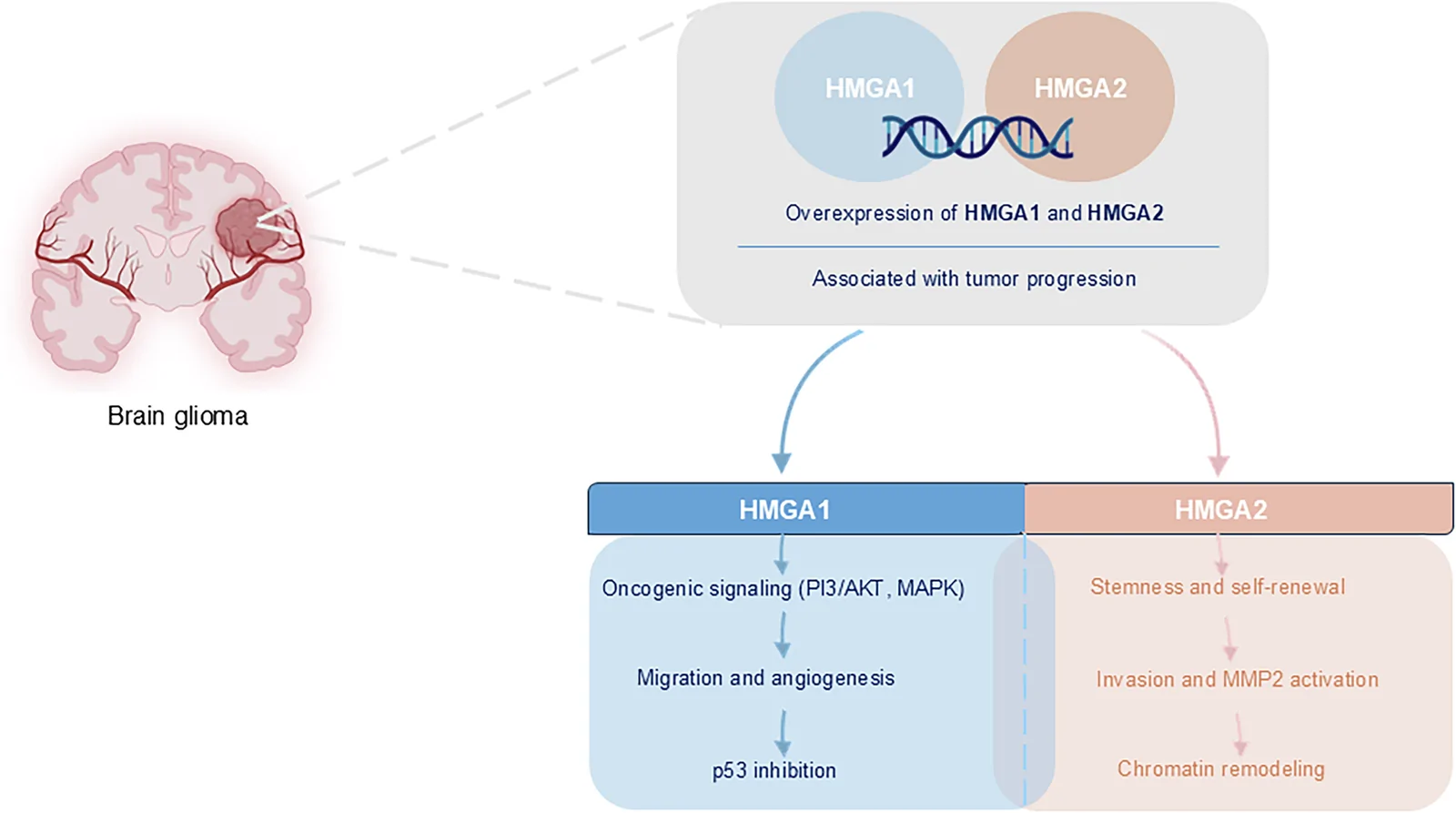
HMGA1 and HMGA2 functions in gliomas.

In conclusion, Fig. 3 highlights the complementary roles of HMGA1 and HMGA2 in glioma biology: HMGA1 mainly drives proliferation, survival under stress, invasion, angiogenesis, and the establishment of an immune-suppressive TME; whereas HMGA2 predominantly maintains a stem-like, therapy-resistant cell population. Moreover, both proteins contribute to tumor heterogeneity, recurrence, and poor prognosis of GBM [70].

### HMGA proteins in pediatric brain tumors: medulloblastoma and atypical teratoid/rhabdoid tumors

HMGA proteins have been implicated also in the biology of pediatric brain tumors such as MB and atypical teratoid/rhabdoid tumors (ATRT), in which they regulate stemness, chromatin dynamics, and invasive behavior. Similarly to what has been observed in other tumors, also in this case HMGA dysregulation has been associated with transcriptional programs that sustain tumor propagation and therapy resistance, exerting context-dependent roles specific to each tumor type.

MB is the most common malignant pediatric brain tumor, arising in the cerebellum. It encompasses four molecular subgroups—Wingless/Integrated (WNT)-activated, Sonic Hedgehog (SHH)-activated, Group 3, and Group 4—each with distinct genetic and epigenetic profiles, clinical outcomes, and therapeutic responses [71]. Recent studies have highlighted HMGA1 overexpression in MB, frequently associated with regional copy-number gain of chromosome 6p21.33–6p21.31, rather than focal amplification of the *HMGA1* locus, and correlated with a high proliferative index and poor prognosis [72].

Functionally, HMGA1 has been implicated in promoting cell cycle progression and invasion through direct transcriptional activation of downstream effectors such as CDC25A, with evidence showing that physical interaction between HMGA1 and the CDC25A promoter is required for transcriptional upregulation [72]. Nevertheless, restricting its function to this single target likely underestimates the broader transcriptional reprogramming ability of HMGA1, which in other tumor types has been shown to cooperate with oncogenic pathways including NF-κB and MYC. Therapeutically, pharmacological inhibition of HMGA1 using minor groove binders such as netropsin has been reported to decrease CDC25A expression and reduce intracranial MB growth in preclinical models [72]. Moreover, although netropsin and other HMGA-interfering natural products have shown in vitro activity, lack of specificity, off-target toxicity, and suboptimal pharmacokinetic properties substantially limit their translational suitability. Indeed, at present, no truly HMGA1-selective inhibitor is clinically available, and pharmacological targeting of HMGA1 remains to be still to investigate [73].

Another key downstream effector of HMGA1 is collapsin response mediator protein 1 (CRMP1), which functions as an invasion suppressor. HMGA1 binds to the CRMP1 promoter and represses its transcription, and HMGA1 depletion leads to increased CRMP1 expression, resulting in inhibition of MB cell proliferation, migration, invasion, and filopodia formation [74].

Finally, although HMGA1 overexpression has been associated with poor outcomes, it is still not clear whether HMGA1 may be considered an independent prognostic factor or whether it reflects co-occurrence with other high-risk features, such as MYC amplification or isochromosome 17q in MB. Further studies across large cohorts, as well as functional validation in subgroup-specific models, are required to define the precise contribution of HMGA1 to MB biology and to evaluate its potential as a biomarker or therapeutic target [72]. In this perspective, proteomic studies have further refined our understanding of HMGA expression in MB. In fact, quantitative proteomics and transcriptomics analysis of MB tissue samples across molecular subgroups revealed differential abundance of proteins, including subgroup-specific isoforms, and highlighted a poor correlation between mRNA and protein levels [75]. Notably, this discrepancy may reflect subgroup-specific post-transcriptional regulatory mechanisms. In particular, differential microRNA (miRNA) expression profiles, previously shown to vary significantly among MB molecular subgroups [72], could contribute to fine-tuning HMGA1 translation and/or protein stability. Indeed, HMGA1 has been identifies as a direct target of miR-124a in MB [72] and is known to be regulated by multiple miRNAs in other tumor types, suggesting that variability in the miRNA landscape across MB subgroups may underlie, at least in part, the uncoupling between transcript abundance and protein expression.

Importantly, HMGA proteins appear to play a relevant role in the most aggressive MB subtype, Group 3, which is frequently driven by MYC amplification and overexpression [75]. Specifically, proteomic and genomic analyses performed separately for each MB subgroup identified two HMGA1 splice isoforms -HMGA1a and HMGA1b- as being preferentially expressed in Group 3 MB, where their abundance correlates with MYC activity and adverse outcome. These findings suggest that isoform-specific regulation of HMGA1 may contribute to the distinctive transcriptional and metabolic programs that sustain Group 3 tumor aggressiveness. This raises the possibility that HMGA1 may cooperate with MYC to promote tumor initiation, proliferation, and metabolic adaptation. Experimental models indicate that astrocyte progenitors can be transformed by MYC to give rise to Group 3 MB. In these tumors, HMGA-mediated pathways may interact with MYC-driven metabolic reprogramming, including upregulation of lactate dehydrogenase A (LDHA), to enhance tumor cell proliferation and survival [76].

Although HMGA2 has been extensively studied in several solid tumors and is widely recognized as an oncofetal protein, its specific role in MB remains less well characterized compared to HMGA1. In fact, in MB, HMGA2 expression has been reported in a single paper in more aggressive molecular subgroups, where it may contribute to maintain tumor cell plasticity, self-renewal capacity, and invasive potential, although direct mechanistic studies remain limited [77]. Future work is needed to clarify whether HMGA2 plays a driver role in MB biology or primarily acts as a marker of undifferentiated, high-risk tumor states.

As far as the role of HMGA proteins in ATRT is concerned, no studies to date have reported a functional involvement of HMGA1 in ATRT, and its potential contribution to ATRT therefore remains unexplored. However, emerging evidence indicates that HMGA2 plays a critical role in this tumor type. ATRTs are highly aggressive pediatric brain tumors characterized by biallelic inactivation of the SWI/SNF-related matrix-associated actin-dependent regulator of chromatin subfamily B member 1 (SMARCB1) tumor suppressor gene. Despite this hallmark event, ATRTs show substantial molecular and clinical heterogeneity and have been stratified into three molecular subgroups: ATRT-TYR (Tyrosinase), ATRT-SHH (Sonic Hedgehog), and ATRT-MYC, each with distinct transcriptional programs, epigenetic signatures, and clinical features [78]. Multiple studies have demonstrated that HMGA2 is frequently overexpressed in ATRT. Zhang et al. showed that in up to 83% of patient samples, HMGA2 protein is elevated, often in concomitance with reduced expression or genomic loss of the tumor suppressor miRNAs let-7a3/let-7b. In fact, reduced let-7 levels are inversely correlated with HMGA2 mRNA expression, and functional assays in G401 rhabdoid cells revealed that reintroduction of let-7 or knockdown of HMGA2 suppresses proliferation, colony formation, and almost completely abrogates invasive capacity. These experiments provided strong evidence that HMGA2 acts as a key oncogenic driver in ATRT [79]. Further functional validation was provided by Kaur et al., who used lentiviral shRNA to stably silence HMGA2 in several ATRT cell lines. HMGA2 knockdown reduced cell growth, proliferation, and clonogenicity while increasing apoptosis, as shown by cleaved caspase-3 and PARP accumulation. Importantly, in vivo studies with orthotopic xenografts demonstrated that suppression of HMGA2 more than doubled median survival in mice (from 58 to 153 days), underscoring its essential role in tumor maintenance and aggressiveness [80].

Notably, the role of HMGA2 may differ across ATRT subgroups, with potential interactions with MYC-driven pathways in the ATRT-MYC subtype, reminiscent of HMGA-MYC cooperation in aggressive medulloblastoma Group 3 tumors, whereas different transcriptional networks may modulate and be modulated by HMGA2 in ATRT-SHH or ATRT-TYR subgroups [81]

Finally, HMGA proteins have been found to be overexpressed also in peripheral nerve sheath tumors (MPNSTs), which are sarcomas originating from Schwann cells. In fact, immunohistochemical analyses have demonstrated that a significant majority of MPNST cases exhibit moderate to strong nuclear staining for both HMGA1 and HMGA2 proteins, distinguishing them from other sarcomas such as synovial sarcomas [82]. Furthermore, HMGA2 has been shown to regulate autophagy pathways in the context of MPNSTs, suggesting that HMGA2 may influence tumor cell survival and response to stress through modulation of macroautophagy [83].

Altogether, these data clearly indicate that HMGA proteins are actively involved in the pathogenesis of several brain cancer types in which they exert an oncogenic activity by enhancing stem-like features, cell proliferation, survival and invasiveness, and their overexpression correlates with poor prognosis and resistance to therapies.

### HMGA proteins in pituitary adenomas

Pituitary adenomas are common intracranial neoplasms arising from the anterior pituitary gland [84]. They are typically benign and slow-growing, but their clinical relevance stems from dysregulated hormone secretion and mass effects on surrounding structures, most notably the optic chiasm and hypothalamus [85, 86]. Based on the capacity to synthetize and release hormones, pituitary adenomas are classified as functioning or non-functioning, with the functioning ones being able to secrete excess anterior pituitary hormones, such as prolactin, growth hormone, or ACTH, leading to well-defined clinical syndromes including hyperprolactinemia, acromegaly, and Cushing’s disease [87]. From a molecular perspective, pituitary adenomas display heterogeneous genetic and epigenetic alterations that influence tumor behavior and lineage differentiation. Dysregulation of TFs critical for pituitary development, chromatin-remodeling proteins, and cell-cycle regulators contributes to their pathogenesis [88, 89]. Increasing evidence highlights the role of chromatin architectural proteins, including HMGA family members, in modulating transcriptional networks and promoting tumorigenic processes within the pituitary gland [90, 91]. Although generally non-malignant, pituitary adenomas can exhibit invasive growth patterns, emphasizing the need to understand the molecular pathways that drive their initiation, progression, and heterogeneity.

In this context, the overexpression of HMGA2 has been shown to actively drive tumorigenesis, as demonstrated by increased HMGA2 levels in human pituitary adenomas and the development of such tumors in HMGA2 transgenic mice [84, 92]. In particular, HMGA2 overexpression occurs in the majority of cases of non-functioning pituitary adenomas (NFPA), despite a low frequency of chromosomal gains or rearrangements, suggesting alternative activating mechanisms [93]. Moreover, it has been observed a correlation between the amplification of the *HMGA2* locus and the increase in HMGA2 levels in human prolactinomas [90]. A mechanistic model links this to enhanced activity of the cell cycle regulator E2F1 because HMGA proteins remove HDAC1 from the pRB/E2F1 inhibitory complex, thereby enhancing E2F1 acetylation and transcriptional activity. This model is strengthened by genetic rescue of the adenoma phenotype when HMGA2 transgenic mice are crossed with E2F1 knockout animals [90, 94]. Consistent with this model, HMGA proteins directly bind to the *CCNB2* promoter and induce *CCNB2* overexpression in both mouse and human pituitary adenomas of different histotypes, further supporting the relevance of HMGA deregulation in the control of the G2/M cell cycle phases [95]. In addition to their effects on cell-cycle regulators, HMGA proteins also promote pituitary tumorigenesis by modulating lineage-specific transcription factors. In particular, they have been shown to induce the expression of the pituitary transcription factor PIT1 (POU1F1), whose up-regulation contributes to pituitary tumor development [96].

However, the mechanistic landscape remains incomplete because additional HMGA dependent routes have been proposed. These include altered interleukin-2 (IL-2) and IL-2 receptor expression, a possible induction of AP-1 activity due to increased expression of the AP-1 components FRA-1 and JunB, and modulation of the pituitary-specific TF Pit-1 [90]. These observations raise the possibility that HMGA overexpression may act through multiple non mutually exclusive axes.

In clinical specimens, HMGA2 expression relates to invasive growth, higher adenoma grade, larger tumor size, increased proliferation index and reduced let-7 expression. Moreover, it has been reported an inverse correlation between HMGA2 and let-7 that supports a post transcriptional route to HMGA2 overexpression in vivo [97]. Importantly, miRNA-based repression of both HMGA1 and HMGA2 appears to be attenuated in pituitary adenomas because a panel of miRNAs targeting both paralogs is downregulated across adenoma histotypes, including non-functioning tumors in which HMGA2 amplification is absent, confirming that loss of post-transcriptional control can be sufficient to increase HMGA levels [98]. This is coherent with clinical data showing that HMGA1 nuclear expression is significantly higher in invasive adenomas and positively correlates with proliferation [99] and with evidence that HMGA2 nuclear abundance is enriched in invasive disease and macroadenomas [100]. In the broader context of brain tumors, this integration of chromatin architecture changes with miRNA deregulation illustrates how lineage-restricted developmental transcriptional programs can be subverted to generate proliferative gain in the pituitary gland, yet whether this HMGA effect can be extended to other brain tumors remains unexplored.

## Hmga proteins as regulators in neurodegenerative diseases

In addition to the well-characterized role of HMGA proteins in neurodevelopment and tumorigenesis, a significative involvement of HMGA proteins in the pathogenesis of neurodegenerative diseases is gradually emerging. First of all, HMGA proteins have been implicated in neuronal plasticity, stress response, and the maintenance of genomic stability [21], processes that are often disrupted in neurodegenerative conditions [101, 102]. Moreover, recent studies, which will be discussed in this section, indicate that aberrant HMGA expression or activity may contribute to neurodegeneration by influencing the accumulation of misfolded proteins, neuroinflammation, and apoptotic cell death.

### HMGA proteins in Parkinson’s disease

Parkinson’s disease (PD) is a progressive neurodegenerative disorder whose clinical phenotype stems primarily from loss of dopaminergic neurons in the substantia nigra and consequently dopamine depletion in the striatum [103]. PD includes both sporadic and familial forms of the disease, in which age-related factors, genetic background, and intrinsic cellular susceptibility collectively contribute to shaping disease onset, progression, and clinical manifestations [103]. At neuropathological level, main hallmark of both sporadic and familial PD is the accumulation of misfolded α-synuclein aggregates within Lewy bodies. Notably, mitochondrial bioenergetic failure, redox imbalance, and impaired lysosomal–autophagic flux seem to contribute to PD pathogenesis [104–106].

Recent evidence revealed a complex and highly context-dependent role of HMGA proteins in PD, enlightening the interplay between chromatin remodeling, non-coding RNAs, autophagy, and oxidative/inflammatory stress pathways. The role of HMGA1 was initially investigated in experimental models of PD induced by exposure to neurotoxins such as 1-methyl-4-phenylpyridinium (MPP+) in vitro and 1-methyl-4-phenyl-1,2,3,6-tetrahydropyridine (MPTP) in vivo. Interestingly, MPP+-treated dopaminergic cell line MN9D and in MPTP-treated C57BL/6 mice, HMGA1 expression is increased in a time-dependent manner [107]. In these models, HMGA1 sustains late-stage autophagic flux and exerts a neuroprotective effect. In particular, HMGA1 transcriptionally activates the expression of miR-103/107, which are critical regulators of the end-stage of autophagy [107]. Moreover, these miRs establish a negative feedback loop directly targeting HMGA1 mRNA, and they restrain the CDK5R1/CDK5 pathway, a known driver of MPTP/MPP+-induced autophagy blockade [107]. Consistently, genetic suppression of HMGA1 exacerbates dopaminergic cell death by further impairing the lysosomal degradation step of autophagosomes, highlighting HMGA1 as a compensatory factor that preserves cellular clearance under neurotoxic stress [107], and as an important regulator of the autophagic flux [108]. However, another study suggests opposite functional output of HMGA1 in both MPP+-treated SH-SY5Y neuroblastoma cells and MPTP-treated mice, in which it is reported to be overexpressed as a consequence of the upregulation of the circular RNA (circRNA) hsa_circ_0054220 [109]. This circRNA acts as a competing endogenous RNA (ceRNA) sequestering two HMGA1-targeting miRNAs, miR-145 and miR-625, thus driving HMGA1 overexpression. This circ_0054220-dependent HMGA1 upregulation promotes neuronal apoptosis and motor deficits, whereas HMGA1 inhibition or circ_0054220 silencing confers neuroprotection [109]. These data strengthen the involvement of HMGA1 in PD pathogenesis, while also pointing to a complex and potentially context-dependent functional role that remains to be fully elucidated.

In addition, HMGA1 may intensify dopaminergic vulnerability by driving inflammatory and oxidative stress responses [110]. Specifically, in MPP+-treated MN9D dopaminergic cells, HMGA1 upregulation enhances the expression of Insulin Receptor Substrate 2 (IRS2), which in turn increases the release of pro-inflammatory cytokines (IL-1β, IL-6, TNF-α) and increases Reactive Oxygen Species (ROS) and Malondialdehyde (MDA) levels, while reducing antioxidant defenses such as Superoxide Dismutase (SOD) activity [110]. Notably, HMGA1 silencing mitigates these deleterious effects, whereas IRS2 overexpression abolishes the protective effect of HMGA1 silencing, pinpointing the HMGA1–IRS2 axis as a critical mediator of injury neuroinflammation and oxidative damage in PD [110]. Altogether, these observations indicate that the phenotypic effects of HMGA1 are not uniform across PD models, reflecting an intrinsic “dual” role of HMGA1.

The divergent outcomes appear to emerge from the specific regulatory networks to which HMGA1 becomes wired; for example, miR-103/107–driven autophagy compensation versus circ_0054220-mediated HMGA1 overamplification or IRS2-dependent inflammatory/oxidative signaling. In other words, it is likely that whether HMGA1 contributes to cytoprotection or exacerbates neuronal injury is critically determined by which stress-response module is engaged (autophagy vs. inflammation/oxidative stress) critically determines.

Differently from HMGA1, HMGA2 is significantly down-regulated in both MPTP-treated mice and MPP+-treated SH-SY5Y cells, and its downregulation is associated with increased neuronal apoptosis [111, 112]. Indeed, HMGA2 overexpression mitigates cell death, confirming the neuroprotective role of HMGA2. In particular, miRNA let-7b-5p has been identified as a direct post-transcriptional regulator of HMGA2. Upregulation of let-7b-5p in PD models promotes apoptosis, while its inhibition rescues neuronal survival, with the latter effect abrogated by HMGA2 silencing [111]. Furthermore, a novel regulatory axis involves the circular RNA circDLGAP4. In SH-SY5Y cells treated with MPP+, circDLGAP4 is downregulated; however, its experimental overexpression has been shown to counteract neuronal injury. CircDLGAP4 interacts with the RNA-binding protein EIF4A3 to enhance HMGA2 mRNA stability and expression, attenuating apoptosis, oxidative stress (ROS, MDA), and inflammatory cytokine release (IL-1β, IL-6, TNF-α). These converging lines of evidence demonstrate that HMGA2 functions as an important protective factor in PD, with multiple layers of regulation reinforcing its neuroprotective activity [112].

Taken together, all these data reveal a relevant role for HMGA proteins in PD pathogenesis. First, HMGA2 shows a consistently neuroprotective profile, with both circRNA-mediated stabilization and miRNA-regulated expression converging to preserve neuronal viability. Second, HMGA1 exhibits dualistic behavior: protective when supporting autophagy, yet potentially pathogenic when dysregulated by ceRNA networks. Third, these findings highlight the central role of non-coding RNAs, including circDLGAP4, hsa_circ_0054220, and various miRNAs (let-7b-5p, miR-103/107, miR-145/625), as critical modulators of HMGA protein activity and, consequently, dopaminergic neuron survival.

From a therapeutic perspective, these insights suggest that context-specific modulation of HMGA proteins could represent a valuable strategy to influence neuronal survival pathways in PD. Enhancing HMGA2 activity, or stabilizing its DNA binding by using pharmacological agents, may reinforce neuroprotective mechanisms such as the maintenance of chromatin architecture, transcriptional programs for neuronal resilience, and autophagy-related processes. Conversely, therapeutic interventions for HMGA1 must be finely tuned; the goal is to specifically suppress its pathogenic overexpression while carefully preserving its essential role in supporting autophagy. Such a targeted approach would address disease-specific dysregulation without broadly perturbing cellular homeostasis. The development of such strategies would require careful consideration of the upstream circRNA and miRNA networks, as well as careful consideration of the disease stage and cellular stress environment. However, besides important insights on functional link between HMGA proteins and PD, further studies are necessary to better shed light on the complexity of their cross-talk and to translate these molecular insights into effective interventions for PD.

### HMGA proteins in Alzheimer’s disease

Alzheimer’s disease (AD) is a progressive neurodegenerative disorder characterized by cognitive decline, synaptic dysfunction and selective neuronal loss, particularly in hippocampal and associative cortical networks. Neuropathologically, AD is defined by extracellular amyloid-β (Aβ) plaque deposition and intracellular neurofibrillary tangles composed of hyperphosphorylated tau. The vast majority of cases are sporadic and multifactorial, with ageing, metabolic dysregulation and environmental stressors (including hypoxia) acting on a genetically susceptible background perturbing proteostasis, synaptic homeostasis and RNA processing [113].

In sporadic AD, HMGA1a has emerged as a critical regulator of aberrant pre-mRNA splicing, particularly affecting the presenilin-2 (*PS2*) transcript. Under physiological conditions, correct splicing of PS2 ensures the production of a functional protein involved in the regulation of the γ-secretase complex, which plays a central role in amyloid-β (Aβ) generation. However, in human neuronal cell lines SK-N-SH and in *post-mortem* AD brain tissues, HMGA1a has been shown to bind to an AT-rich sequence motif located upstream of exon 5 in PS2 pre-mRNA [114], thus preventing the recruitment of the U1 small nuclear ribonucleoprotein (snRNP) 70 K subunit to the 5′ splice site. As a consequence, exon 5 is skipped, leading to the production of the pathogenic isoform PS2V [115]. In turn, this increases the susceptibility of neurons to endoplasmic reticulum (ER) stress, facilitating apoptotic cell death from one side [116], and to the other enhances the accumulation of Aβ peptides, the key drivers of amyloid plaque deposition [117]. Importantly, this aberrant splicing event appears to be particularly pronounced under hypoxic conditions, a known environmental risk factor for AD. In hypoxia, HMGA1a accumulates in nuclear speckles enriched in the splicing factor SC35, suggesting a dynamic interaction between HMGA1a and splicing regulators that amplifies the pathological misprocessing of PS2 [114]. These findings indicate that HMGA1a, beyond its canonical role in chromatin architecture, acts as a sequence-specific RNA-binding protein capable of redirecting splicing pathways toward a disease-promoting outcome. This functionality highlights the complexity of HMGA1a role in cellular homeostasis, and positions it as a new potential therapeutic target to modulate PS2 splicing and mitigate PS2V-associated neurotoxicity in sporadic AD.

More recently, an alternative HMGA1-dependent mechanism has been proposed for tauopathies, a heterogeneous group of neurodegenerative diseases characterized by progressive cognitive, motor, and neuropsychiatric decline due to accumulation and aggregation of the tau protein in neuronal and glial cells [118]. In fact, HMGA1 was identified as a transcriptional repressor of *MAPT*, the gene encoding the tau protein, and it negatively regulates its expression in human neuronal cell models, primary mouse cortical neurons, and *Hmga1*-deficient mice. Accordingly, reduced HMGA1 expression led to increased MAPT transcription and accumulation of total and phosphorylated tau species, including pathogenic phospho-tau forms associated with neurodegeneration. HMGA1 deficiency was also accompanied by reduced expression of the insulin receptor (INSR) and the neuronal glucose transporter GLUT3, together with impaired cerebral glucose uptake, supporting a link between tau pathology and altered brain energy metabolism [119]. Importantly, the HMGA1 rs146052672 loss-of-function variant, previously linked to insulin resistance and type 2 diabetes, was found to be enriched in patients with tauopathy, and associated with a more severe clinical progression. These findings support HMGA1 deficiency as a mechanistic bridge between tau pathology and metabolic dysfunction, expanding HMGA1 pathogenicity in AD beyond PS2 alternative splicing alone [119].

Besides HMGA1, HMGA2 may also contribute to AD disease pathogenesis. It is normally expressed at very low levels in the adult brain, but its aberrant upregulation has been reported in AD experimental models. For instance, in a mouse model in which AD is induced by scopolamine injection, HMGA2 expression was significantly increased in hippocampal tissue and was associated with cognitive impairment, neuroinflammation, and oxidative stress [120]. Consistently, functional silencing of HMGA2 resulted in improved learning and memory performance, reduced inflammatory cytokine production (TNF-α, IL-6, IL-1β), attenuation of oxidative stress markers, and amelioration of hippocampal injury. Mechanistically, HMGA2 appears to exert a detrimental role by suppressing the neuroprotective PI3K/AKT signaling pathway, whose activity is known to regulate neuronal survival, mitochondrial integrity, and amyloid-related metabolic processes [120]. Importantly, HMGA2 was identified as a direct target of miR-132, a miRNA critically involved in synaptic plasticity and neuronal homeostasis. In AD mice, miR-132 levels were markedly reduced, concomitant with HMGA2 upregulation, whereas restoration of miR-132 or direct HMGA2 silencing reactivated PI3K/AKT signaling and conferred neuroprotection. These findings suggest that dysregulation of the miR-132/HMGA2 axis contributes to AD-associated neurodegeneration and positions HMGA2 as a negative modulator of neuronal resilience. Together with HMGA1-mediated alterations in RNA splicing and tau regulation, these data support a broader involvement of HMGA family proteins in AD pathogenesis, acting through complementary mechanisms that converge on neuronal stress responses, synaptic dysfunction and cognitive decline. Collectively, findings related to both PD and AD support the idea that HMGA proteins act as highly context-dependent regulators in neurodegeneration. Their effects appear to vary according to the specific cellular subtype, brain microenvironment, disease stage and molecular pathways involved, resulting in either neuroprotective or pathogenic outcomes.

### HMGA proteins in spinocerebellar ataxias and ataxia-telangiectasia

Spinocerebellar ataxia (SCA) refers to a genetically heterogeneous group of progressive neurodegenerative disorders, primarily affecting the cerebellum and its afferent/efferent pathways, many of which are caused by CAG trinucleotide repeat expansions leading to polyglutamine-containing proteins. Among them, spinocerebellar ataxia type 1 (SCA1) is caused by an expanded polyglutamine tract in the ataxin-1 (ATXN1) protein, whose expression levels critically influence disease onset and severity. Clinically, SCA is characterized by gait instability, limb and ocular dysmetria, impaired motor coordination and, in many subtypes, cognitive and psychiatric involvement. Despite distinct genetic etiologies, convergent molecular pathways have emerged as SCA drivers, including transcriptional dysregulation, RNA toxicity, proteostasis collapse and selective vulnerability of Purkinje cells. Maladaptive stress responses, impaired axonal transport and synaptic dysfunction contribute to progressive cerebellar degeneration, placing SCA within a broader framework of expansion-associated disorders in which mis-processing of RNA/proteins drives neuronal demise [121].

Although HMGA proteins have not been directly implicated in the pathogenesis of SCAs, indirect evidence suggests a potential link between HMGA1, HMGA2 and SCA-related molecular pathways. For instance, HMGA1 can positively regulate the expression of specific miRNAs, including miR-101b and miR-196a-2, in mouse embryonic fibroblasts (MEFs) and in several mouse tissues. Notably, miR-101b was identified as a direct post-transcriptional regulator of the ATXN1 gene, which plays a central role in neural homeostasis by modulating transcriptional repression and RNA metabolism in Purkinje cells [122]. HMGA1-dependent expression of miR-101b results in reduced ATXN1 protein levels without affecting ATXN1 mRNA abundance, indicating a mechanism of miRNA-mediated translational repression [122]. This finding is particularly relevant in the context of SCA1, as ATXN1 protein dosage has been shown to modulate neurotoxicity and disease progression. In addition, HMGA1 also regulates miR-196a-2, which directly targets HMGA2 [122]. Although HMGA2 has not been directly associated with SCAs, its involvement in neural progenitor maintenance and chromatin regulation suggests that HMGA1–miRNA–HMGA2 regulatory loops may influence neuronal homeostasis and vulnerability under pathological conditions. These findings support the hypothesis that HMGA1 may indirectly participate in SCA-related molecular networks by modulating miRNA expression and, consequently, ATXN1 protein levels. While current evidence does not demonstrate a direct causal role for HMGA proteins in SCA pathogenesis, the HMGA1–miR-101b–ATXN1 axis represents a biologically mechanism linking chromatin architecture, post-transcriptional regulation and neurodegeneration. Further studies in neuronal and in vivo models will be required to clarify whether dysregulation of HMGA proteins contributes to disease onset or progression in SCAs.

Besides their possible involvement in SCA, HMGA proteins are implicated also in ataxia-telangiectasia (AT), a neurodegenerative disorder marked by progressive cerebellar atrophy and marked Purkinje cell vulnerability [123]. AT is caused by loss-of-function mutations in the ATM (Ataxia-Telangiectasia Mutated) kinase, a master regulator of the DNA damage response [124]. In this context, HMGA proteins emerge as important ATM substrates: following genotoxic stress, ATM phosphorylates HMGA proteins, modulating their chromatin-binding properties. Specifically, HMGA1 is phosphorylated by ATM at serine 88 within a conserved SQ motif, whereas HMGA2 contains a corresponding SQ motif at serine 102, also targeted by ATM [125]. This post-translational regulation enhances the transcriptional activity of HMGA proteins, including their ability to activate ATM expression, thus amplifying the DNA damage response through a positive feedback loop. Initially characterized in tumor cells, this loop promotes survival under genotoxic stress, suggesting a conserved mechanism by which HMGA proteins support cellular homeostasis [125]. Although these dynamics have been characterized predominantly in non-neuronal systems, mainly in MEFs, they highlight a principle with clear neurobiological relevance: HMGA proteins participate in core stress-response pathways essential for the integrity of long-lived cerebellar neurons.

Hence, while HMGA involvement in SCAs arises from mechanisms such as miRNA-mediated regulation of *ATXN1*, and in AT is linked to ATM-dependent DNA damage responses, both contexts underscore the potential contribution of HMGA proteins to cerebellar vulnerability through distinct yet complementary routes.

Future studies determining whether analogous HMGA–ATM interactions occur in neuronal populations, and particularly within susceptible cerebellar cell types, will be crucial to fully elucidate their role in cerebellar neurodegeneration.

**Fig. 4:**
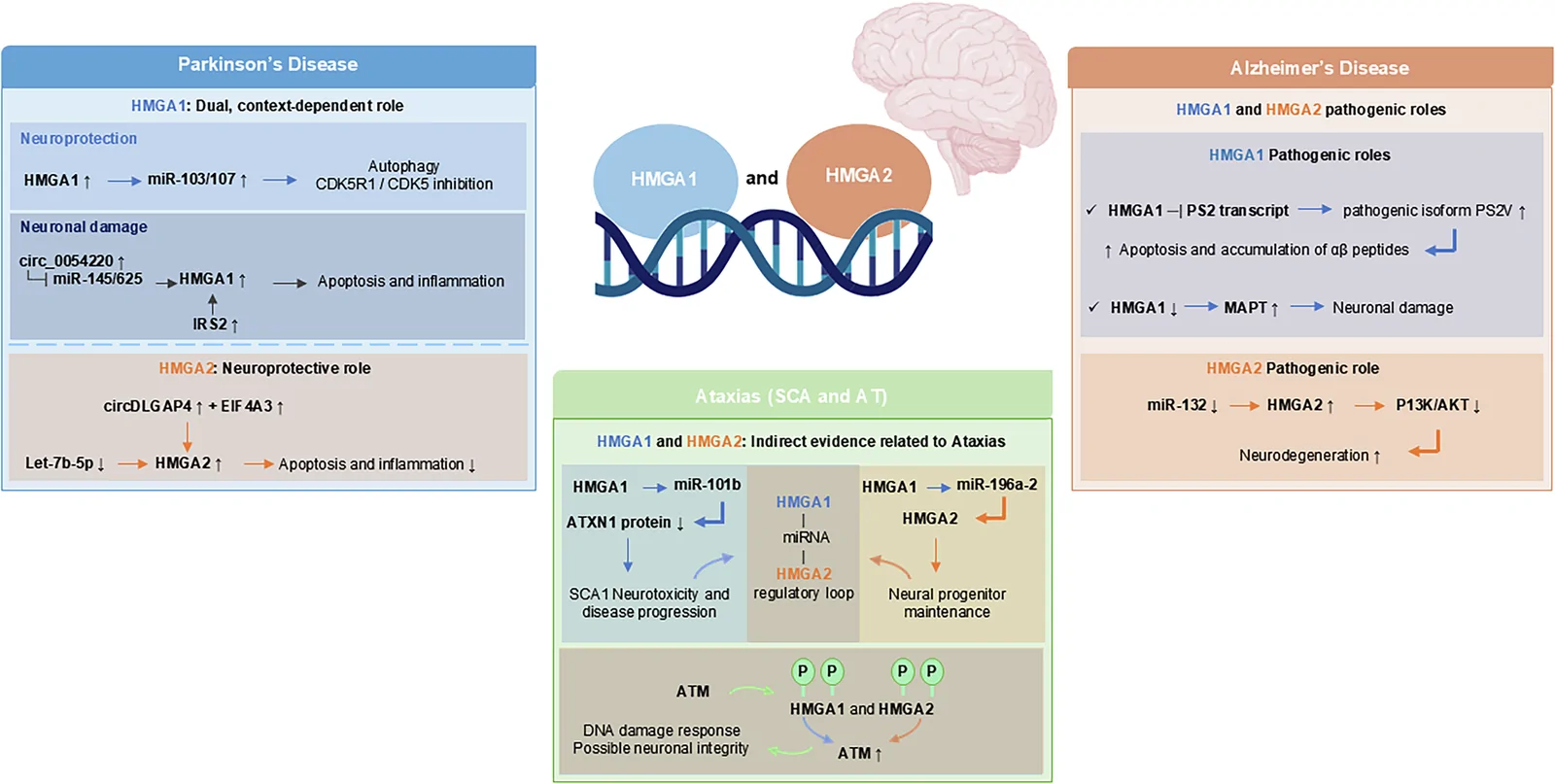
HMGA proteins as regulators in neurodegenerative diseases.

Figure 4 illustrates how HMGA proteins are involved in the regulation of the described neurodegenerative diseases, highlighting their context-dependent effects.

## Perspectives

The critical role of HMGA proteins in the physiopathology of the nervous system make them novel promising therapeutic targets. However, their pleiotropic functions and cell-context dependence represent a major limitation for their therapeutic translation. Selective tool able to finely modulate their expression and/or activity remains the major challenge for their actual use in clinical practice, for both oncological and neurodegenerative diseases. Promising results emerged from experimental approaches are based on RNA interference or restoration of tumor-suppressive miRNAs (e.g., let-7, miR-1297, miR-296-5) to reduce HMGA expression. These approaches showed efficacy in reducing glioma stemness, invasion, and chemoresistance in preclinical models, but clinical studies are still lacking.

However, the clinical translation of RNA-based therapeutic strategies in brain tumors remains particularly challenging due to the presence of the blood–brain barrier (BBB), which significantly limits the delivery, stability, and bioavailability of RNA-based molecules within the central nervous system.

Despite the significant advances in nanomedicine and nanotechnology over the last few years, transitioning from a promising scientific field to a cornerstone of clinical reality, crossing the blood–brain barrier (BBB) remains the major challenge in the treatment of brain diseases. Fortunately, there has been progress in the development of nanomedicine-based delivery systems [126]. In order to enhance brain parenchyma accumulation, these nano-platforms are increasingly functionalized with targeting ligands (such as transferrin, apolipoproteins, or cell-penetrating peptides) that exploit receptor-mediated transcytosis pathways across the brain endothelial cells [127]. While preclinical studies are very promising, transforming these platforms into routine clinical practice presents a multi-tiered challenge: achieving reproducible large-scale manufacturing (scalability), avoiding entrapment and degradation within the endo-lysosomal compartments of brain endothelial cells, and ensuring long-term biocompatibility without neurotoxic side effects. Addressing these limitations will be essential for the successful implementation of HMGA-targeting approaches in patients.

Pharmacological inhibition of HMGA–DNA interactions with minor groove binders, as well as strategies combining HMGA2 targeting with MYC inhibition in ATRT, represent additional avenues to pursue, although the assessment of their specificity and toxicity is fundamental for their translational potential.

Approaches such as restoring miRNA-mediated repression, targeting upstream oncogenic pathways, or designing small molecules that interfere with HMGA–DNA or HMGA–protein interactions remain promising, but studies are still preliminary. Moreover, the emerging role of HMGA proteins in neurodegenerative disorders suggests that epigenetic therapies aimed at restoring chromatin homeostasis may also represent a potential future direction. Future studies will be required to better define context-specific HMGA functions and to develop therapeutic strategies able to selectively modulate their activity in brain tumors and neurodegenerative diseases.

## Conclusion

The general importance of HMGA proteins in chromatin architecture, transcriptional regulation, embryonic development and cancer biology has been well established over several decades. Recently, growing evidence is accumulating in support of the idea that HMGAs are key determinants of the development and physiopathology of the nervous system. During embryogenesis, HMGA1 and HMGA2 sustain neural stem cell proliferation and plasticity, orchestrating lineage specification and the temporal sequence of neurogenesis. In adult tissues, their repression is crucial to maintain the brain homeostasis, yet residual expression in neurogenic niches underscores their latent role in sustaining neural progenitors throughout life. The pathological reactivation of HMGA-mediated developmental programs in the adult brain, however, constitutes a double-edged sword: while conferring regenerative potential, it also predisposes cells to malignant transformation and aberrant stress responses. Similarly to what has been observed in many other tumor types, HMGA proteins are aberrantly expressed and re-engage developmental transcriptional programs to promote proliferation, invasion, stemness, and therapeutic resistance also in brain tumors, particularly gliomas, medulloblastomas, and ATRTs. HMGA1 predominantly supports proliferation and stress adaptation, whereas HMGA2 sustains stem-like and invasive traits, contributing to tumor heterogeneity and poor clinical outcomes.

Beyond well-established role in oncogenesis, several studies have also highlighted HMGA proteins as critical modulators of neuronal vulnerability in neurodegenerative disease. In AD, HMGA1a functions as a sequence-specific RNA-binding factor that drives aberrant splicing of presenilin-2, contributing to amyloidogenesis, ER stress, and glial-mediated neuroinflammation. In PD, HMGA1 and HMGA2 integrate transcriptional, epigenetic, and post-transcriptional pathways that amplify oxidative stress, mitochondrial dysfunction, and impaired autophagy, thereby influencing dopaminergic neuron survival. Similarly, in SCA, HMGA proteins may converge on DNA damage responses and miRNA networks, linking chromatin plasticity to neuronal integrity. Although mechanistic studies remain limited, accumulating evidence supports a model in which HMGA dysregulation in the adult brain leads to epigenetic reprogramming that promotes neuronal dysfunction and neurodegeneration. This suggests potential roles for HMGA proteins as biomarkers of disease progression and as targets for epigenetic therapies aimed at restoring chromatin homeostasis.

Through their diverse functions, HMGA proteins stand at the crossroads of neural development, cancer biology, and neurodegeneration. Their ability to orchestrate chromatin plasticity and regulatory networks presents both opportunities and challenges for translational applications. Future work should focus on unraveling their context-specific functions, clarifying their contributions to disease heterogeneity, and developing therapeutic strategies that selectively modulate HMGA activity. Approaches such as restoring miRNA-mediated repression, targeting upstream oncogenic pathways, or designing small molecules that interfere with HMGA–DNA or HMGA–protein interactions remain promising but preliminary. Ultimately, a deeper understanding of HMGA biology may not only elucidate the fundamental principles of nuclear architecture but also open new avenues for interventions in brain tumors and neurodegenerative disorders.
